# The weight-loss experience: a qualitative exploration

**DOI:** 10.1186/s12889-016-3045-6

**Published:** 2016-05-04

**Authors:** David Rogerson, Hora Soltani, Robert Copeland

**Affiliations:** Academy of Sport and Physical Activity, Sheffield Hallam University, Collegiate Crescent Campus, Sheffield, S10 2BP UK; Centre for Health and Social Care Research, Sheffield Hallam University, Collegiate Crescent Campus, Sheffield, S10 2BP UK; Centre for Sport and Exercise Science, Sheffield Hallam University, Collegiate Crescent Campus, Sheffield, S10 2BP UK

**Keywords:** Weight loss, Qualitative, Experience

## Abstract

**Background:**

Long-term weight management consists of weight-loss, weight-loss maintenance, and weight-gain stages. Qualitative insights into weight management are now appearing in the literature however research appears to be biased towards explorations of weight-loss maintenance. The qualitative understanding of weight loss, which begets weight-loss maintenance and might establish the experiences and behaviours necessary for successful long-term weight management, is comparatively under-investigated. The aim of this study was to investigate the weight-loss experiences of a sample of participants not aligned to clinical intervention research, in order to understand the weight-loss experiences of a naturalistic sample.

**Methods:**

Participants (*n* = 8) with weight-loss (*n* = 4) and weight-maintenance experiences (*n* = 4) were interviewed using a semi-structured interview to understand the weight-loss experience. Interview data was analysed thematically using Framework Analysis and was underpinned by realist meta-theory.

**Results:**

Weight loss was experienced as an enduring challenge, where factors that assisted weight loss were developed and experienced dichotomously to factors that hindered it. Participants described barriers to (dichotomous thinking, environments, social pressures and weight centeredness) and facilitators of (mindfulness, knowledge, exercise, readiness to change, structure, self-monitoring and social support) their weight-loss goals in rich detail, highlighting that weight loss was a complex experience.

**Conclusions:**

Weight loss was a difficult task, with physical, social, behavioural and environmental elements that appeared to assist and inhibit weight-loss efforts concurrently. Health professionals might need to better understand the day-to-day challenges of dieters in order to provide more effective, tailored treatments. Future research should look to investigate the psycho-social consequences of weight-loss dieting, in particular self-imposed social exclusion and spousal sabotage and flexible approaches to treatment.

## Background

Weight loss is a complex problem, where physical, environmental, and behavioural factors disrupt and assist dieters in their pursuit of negative energy balance [1]. Evidence indicates that weight loss leads to adaptations that increase appetite [2], the desire to eat and preoccupations with food [2], cravings [3], and reduced energy cost of activity [4]. Weight-loss is therefore multifaceted, and often difficult to achieve, explaining why over 80 % of dieters regain lost weight [5]. Small proportions of individuals do achieve and maintain weight loss however [5], and research has sought to identify factors that differentiate these individuals from those who are unable to achieve their weight-loss goals [6–8]. Successful dieters modify their lifestyles to achieve early successes [6]; they maintain and remodel their newfound behaviours over time [7]; they possess social support mechanisms [1, 6–8], and are attentive to threats to their weight status [6, 7]. Importantly, successful dieters view weight loss as a lifelong commitment [1, 6–8].

Despite a growing body of evidence that unpicks factors associated with successful weight management [1, 6–8], much of the literature has been undertaken using quantitative methods, which might not fully reveal the complexity of the experience [8, 9]. The qualitative study of weight management appears to be in its infancy and deep insights into the weight-management journey have begun to appear in the literature [7, 10]. Much of this research has been conducted using samples obtained from weight-management intervention studies [7, 8], often in an attempt to explore weight-loss maintenance [7], and as a result much less is known about the weight-loss phase of the weight-management journey, particularly the experiences of individuals who accommodate weight-loss without the focus of research-based interventions. The aim of this study was to explore the weight-loss experiences of dieters not aligned to research, to provide a rich, detailed account of how these people experienced and accommodated their weight loss efforts, in real-life contexts.

## Methods

### Theoretical underpinning

This study was underpinned by realist meta-theory, which enables and requires the deep exploration and description of participants’ perspectives, experiences and realities in an objective and explicit matter [11]. Ethical approval for this research was granted by Sheffield Hallam University’s research degrees ethics committee.

### Sampling

Participants were included using purposive sampling, and data saturation was used as a guiding principal for sample size, which was determined iteratively [12, 13]. Adverts for participants who had experiences of undertaking a weight-loss diet were placed in local slimming clubs, health clubs and gyms, the employee intranet and email lists at Sheffield Hallam University, and by networking with colleagues. Participants were recruited into the study until data saturation was achieved, which was defined at the point where no new themes emerged from the transcripts [13]. To achieve this, each participant was interviewed and recorded. Recordings were then transcribed and analysed upon receipt, and further participants were recruited to the study up until no new themes emerged from the analyses. Guest and colleagues demonstrated that samples as few as 6 participants were sufficient to achieve data saturation in their investigation to determine non-probabilistic sample size requirements [13]. A sample of 6 participants was therefore expected to be the minimum sampling requirement to achieve data saturation in this research.

### Data collection

Interview data was collected face to face and prompted by an interview guide that was developed from the extant literature. All interviews were recorded using a portable digital recording device (Olympus Digital Voice Recorder, model WS-321 M, Olympus Imaging Corp, China) and were conducted in a neutral, quiet environment that was mutually agreed upon prior to data collection. Interviews were supplemented with field notes [14], and lasted approximately 60–75 min. Respondent validation was sought from each participant after their interviews to ensure credibility [12, 14], and to ensure that the interviews had provided them with sufficient opportunity to articulate their experiences [12, 14]. Upon completion of the interviews the audio files were saved to a password-protected external hard drive (WD My Passport, Western Digital, Irvine, California, USA), under the prefix of participant 1, participant 2, etc., to ensure participant confidentiality and anonymity. All interviews were transcribed *verbatim* by a data transcription service.

### Data analysis

Data was analysed thematically using Framework Analysis [15]. The initial stages of data reduction and interpretation included data familiarisation and cross-referencing the manuscript texts with the aims of objectives of the research [15]. A preliminary theoretical framework was then constructed, and initial codes were identified on a line-by-line and paragraph-by-paragraph basis using open coding [15, 16]. Themes and sub-themes emerged inductively as described by Braun and Clarke [16], and participant’s words were used to generate in-vivo codes to remain true to the data [15, 16]. The original transcripts were then imported into Nvivo 10 (Qualitative Solutions and Research International, Victoria, Australia) to index the data in stage three. Text was transposed from the electronic transcripts into themes and sub-themes which were created as nodes within Nvivo, indexing the data. The Framework Matrices tool in Nvivo was then used to create matrices where each row represented a participant and each column represented a theme or sub-theme. Data for each theme and participant was then summarised in the framework matrices by referring directly to the coded/indexed data from stage three, reducing the data further [15]. Once completed, the framework matrices were exported into Microsoft Excel 2010 (Microsoft Corporation, Redmond, WA) and printed off for interpretation. To interpret the data, themes were triangulated with the summaries, original text and audio files, to allow for the conceptualisation of the data as a whole [15, 17].

### Verification

To enhance dependability and reduce bias [12, 17], the codes, themes and theoretical framework were verified by colleagues prior to the charting of stage 3 and after the mapping and interpretations of stage 5, as a form of peer debriefing [13, 16]. To achieve this, meetings were organised and data was provided to the attendees (who were blind to the results at each stage) prior to each meeting. During the meetings feedback was provided, findings were discussed, and the interpretations and theoretical framework were approved.

### Reflexivity

In an effort to enhance credibility, objectivity and rigour in line with realist research practice [11, 17, 18] the primary author of this research engaged with reflexivity throughout the research process, and the following background information should be used to appraise the credibility of this study [18]. This research question was problematized through work that the primary author undertook to complete a Professional Doctorate, which highlighted that weight-loss-specific data might be lacking from the qualitative weight-management literature. The primary author of this study is a registered Nutritionist with experience of working with weight-loss clients. During the development of this research the primary author, however, sought to separate their experiences from the decisions made during the development, design, and undertaking of the study, such that data collected best represented the participants’ experiences and that data was collected as objectively as possible, which is a necessity in realist research practice [11, 17]. This was achieved through the processes of respondent validation and peer debriefing described earlier. The principal investigator had no prior relationship with any of the participants recruited, who were identified through the recruitment channels discussed. During qualitative interviewing power dynamics might be shifted towards the researcher, which can bias the data [14]. The primary author therefore sought to facilitate information exchange by building rapport, encouraging dialogue, pursuing the interview agenda flexibly, and taking into account and exploring the participants’ responses actively and co-operatively, which is characteristic of realist interviewing, and considered to be good practice [11, 14, 17].

## Results

### Participants

Eight volunteers participated in this study. The participants were British, white citizens, from both genders, and of 40 ± 10 years (M ± SD) of age. Three of the participants (4, 5 and 6) had undertaken a weight-loss education programme within two years of undertaking the interviews. Two participants (6 and 7) were members of a commercial slimming group (Slimming world Ltd, Alfreton, UK). Two participants had previously received consultation services from a registered nutritionist (1 and 2), and one of these participants was a retired athlete (1). Two of the participants had received no dietary education or consultation services (3 and 8) and embarked on their weight-loss journeys without help. Four of the participants were actively trying to lose weight (1, 3, 6 and 7), and four of the participants were maintaining weight loss at the time of data collection (2, 4, 5 and 8). None of the participants were participating in (or had participated in) a weight-loss intervention research study. Participant’s information can be found in Table 1.

**Table 1:** Participants’ information

Participant	Gender	Age	Education level	Relevant information	Goals
1	Female	29	Post-Graduate	Ex-athlete	Weight Loss
2	Male	35	Post-Graduate	Single Male	Weight Maintenance
3	Male	34	Post-Graduate	Working Father	Weight Loss
4	Male	43	Undergraduate	Commercial Programme	Weight Maintenance
5	Female	56	Undergraduate	Commercial Programme	Weight Loss
6	Male	54	Undergraduate	Commercial Programme	Weight Loss
7	Female	36	Post-Graduate	Working mother	Weight Loss
8	Female	34	High School	Single Female	Weight Maintenance

**Fig. 1:**
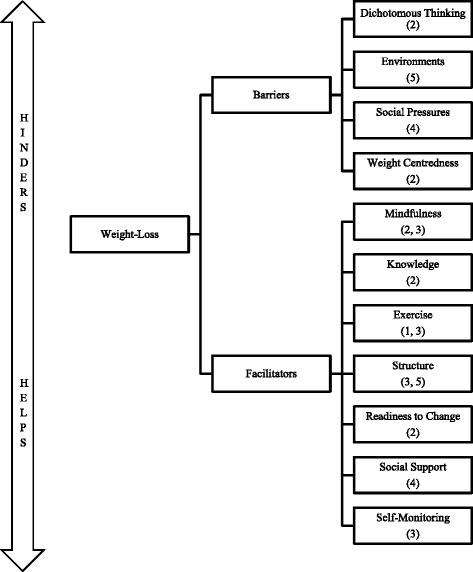
Theoretical Framework. Thematic, theoretical framework depicting participants’ experiences; the arrow represents the dichotomy experienced between barriers and facilitators, demonstrating how barriers hindered the participants’ weight-loss efforts and facilitators helped them. Factors that participants described are categorised and numbered into practical (1), cognitive (2), behavioural (3), social (4) and environmental (5) subgroups, which are themed as barriers to and facilitators of weight loss. In some instances (mindfulness, exercise and structure) these factors belonged to multiple subgroups

The participants described weight loss as an enduring challenge, which could be difficult physically, mentally and emotionally. The participants described (in depth) how losing weight requires the careful and consistent management of factors that support weight loss with those that prompt relapse. These were themed into barriers to and facilitators of weight loss, and were revealed to be complex and multidimensional issues that participants experienced during their journeys. Barriers to weight loss consisted of “dichotomous thinking”, “environments”, “social pressures” and “weight centeredness”. Facilitators of weight loss included “mindfulness”, “exercise”, “structure”, “knowledge”, “readiness to change”, “self-monitoring” and “social support”. A schematic representation of the participant’s weight-loss experiences is provided in Fig. 1 and a description of the themes follows.

### Barriers

#### Dichotomous thinking

All of the participants (*n* = 8) described dichotomous weight-loss experiences, and three participants described themselves specifically as being “*all or nothing*” in personality (1, 4 and 5). These polarized thinking patterns were revealed to rationalise phases of rigid restraint, eating disinhibition and weight cycling, leading to the development of this theme:

“*I spoke to one of my friends and she said, you know, if you’re really craving chocolate just have a small square of chocolate or whatever - there’s no way I could do that. It’s like almost all or nothing*” (1)

The loss of restraint leading to disinhibited eating was a common experience for most participants (*n* = 7: 1, 3–8), and “*switching off*” (1) and “*frames of minds*” (5) were cited as allegories and explanations for the behaviour. Dichotomous thinking appeared to be a common issue for all participants, regardless of whether their goal was weight loss or weight-loss maintenance, and appeared to reflect both goals. While reflecting on her experiences, one participant described that dieting was a difficult psychological and emotional journey that punctuated polarised thinking and behavioural patterns:

“*It’s just in your head, you know, it’s a psychological thing, and sometimes you’re in the zone and sometimes you’re not… it’s as if you’ve got so much emotional energy to give and when you’re stressed out on other things, focusing on the diet’s hard, you know*” (5)

#### Environments

This theme described how the participants’ environments challenged their consistency. The work environment was revealed by most to create problems, and did not appear to be specific to participants with weight loss or weight-loss maintenance goals:

*“Suddenly you’re called away for work to go somewhere else and the food cupboard that you’ve put together, the larder you’ve put together is not available to you*” (6)

“*At the end of the day the healthy foods are the least accessible ones when you’re at work*” (7)

For those that had families, the home could be problematic too, where the presence of forbidden foods and appetite-promoting stimuli created temptations:

“*There were lots of indulgent type treaty foods knocking around the house, whilst I’m not offering up excuses, but that’s sort of tough”* (5)

#### Social pressures

All of the participants, regardless of whether weight loss or weight-loss maintenance was their goal at the time of interview, described a broad range of social issues that impacted their behaviours, and it was clear that socialising and social eating created difficulties. Firstly, some participants (1, 4, 5, 6 and 7) suggested that they became self-aware when eating out, and were conscious of the perceptions of others when eating in company:

“*And a big thing for me is like I feel daft if I ask for like a healthier option because I’m convinced that people will be thinking well you obviously don’t do that all the time*”? (1)

Two participants directly described feeling “*pressured*” and under “*tension*” because their newfound behaviours alienated them from friends and family (5 and 7):

“*There’s always a tension. I think because you are a fat person and you’ve eaten the way fat people eat and you’ve lived the lifestyle that fat people live, and you’ve got an entire network of people around you that are like that. So when you stop being like that, it’s doubly hard*” (5)

The participants also revealed that family members could act as saboteurs (5 and 7), tempting them with foods, despite knowing and understanding their weight-loss goals and challenges:

“*You know, and then she’ll say afterwards shall I defrost you a bit of cake? You know I can’t. You know, or she’ll say shall we go to the chippy? You know I can’t. And little things like that. Well another one won’t hurt you.”* (5)

#### Weight centeredness

Participants (1–4, 6–7) characterised weight loss as an ongoing, enduring task, often at the forefront of their thinking, which created the perception of a weight-centred existence, and appeared to be most pertinent (but not exclusive) to those participants who were seeking weight loss at the time of interview:

‘*At the moment it’s* (weight loss) *an ongoing process and has been for six months.*’ (2)

‘*It’s* (weight loss) *just constantly something that’s on your mind.*’ (7)

Weight centeredness could be both positive and negative in terms of the participants’ weight-loss goals; positive experiences are depicted in the mindfulness theme below; negative experiences are described within this theme. For some (1, 3–6), the development of weight-loss behaviours, such as calorie counting and self-monitoring, led to obsessiveness, which was a negative weight-loss experience:

*‘I was weighing myself several times a day, it became an obsession.’* (4)

For one participant (3), activities like weighing himself became demotivating if weight loss was not experienced as quickly as he would have liked, despite achieving positive weight changes. For this participant, weight centeredness led to unrealistic weight-loss expectations:

‘*I often get demotivated if it doesn’t come down at the rate that I want it to or if for example I might lose two kilos one week and a kilo the week after. For me that becomes slightly de-motivational and I know that it’s a strange thing to say, because that’s still good weight loss.*’ (3)

The participants articulated the need to reduce obsessive thinking and behaviours, recognising that the development of obsessive habits might be unsustainable and abnormal behaviour:

*‘I think for me being more aware of it* (calories) *but not obsessing about it is the key.*’ (1)

‘*This is where the not going so far that I become obsessive about it* (dieting) *comes in.*’ (3)

Maintaining weight-loss behaviours required consistent, focused attention and emotional resources; participants explained that maintaining this focus was challenging, especially when faced with unfortunate life circumstances:

‘*You’ve got so much emotional energy to give and when you’re stressed out on other things, focusing on the diets hard.*’ (5)

Participants revealed that they needed to recommit to their weight-loss goals regularly, despite experiencing unfortunate life-events or a lack of progress, highlighting that weight loss requires persistence and dedication, and that successful weight loss might be experienced as a constant challenge:

“*The hardest thing is that you have to recommit to doing it every day, and that’s hard when you fall off the wagon or you don’t see progress. The ability to stay focused and have the goal in mind becomes that little bit tougher.*” (6)

### Facilitators

#### Mindfulness

All of the participants described cognitive factors that facilitated their weight-loss efforts. For all of the participants, regardless of goal at the time of interview, weight loss facilitated or required the development of meta-cognition:

*“It’s probably a little bit more conscious, in the forefront of my mind of paying attention to where I am, what I’m doing and how I’m managing my diet and behaviour*” (2)

Participants explained that becoming mindful and self-aware allowed them to make conscious eating and behavioural decisions, facilitating weight-loss goals. For some, however, mindfulness was something that needed to be constant, and at the forefront of their thinking, to continually make good eating decisions:

“*But a lot of the time it is just, it’s one of those things that’s always on your mind,* (making good eating decisions) *because you’ve done it for so long* (snacking) *where you’ve just not even thought about it, yeah well I’ll just have another biscuit or whatever.*” (7)

Whilst being reflective and thoughtful was a facilitator of weight loss, one participant (5) recognised that having to be constantly mindful was also a challenge, reflecting the weight-centeredness sub-theme described earlier:

“*And even though you recognise these behaviour patterns, it still don’t stop your brain going into that cycle. So you have to be mindful, you have to consciously choose not to do it. That takes emotional energy, the conscious choice*.” (5)

#### Knowledge

Seven of the participants stated that increasing their knowledge of food and nutrition had been formative (2–8), and improving knowledge appeared to be a key factor in the achievement of weight loss specifically. Participants increased this knowledge in a number of ways, and knowledge was gained via health professionals and/or slimming clubs (2–5), or through their own research (1). Increasing knowledge included understanding science, physiology and food and nutrients (4 and 6):

“*I started to learn about what we needed as fuel and why we needed it”* (6)

Increasing practical knowledge, such as new recipes and food choices, was also described as being valuable (1, 5, 7 and 8):

“*One of the reasons that Slimming World helps because the people there who’ll give you ideas for things to cook*” (4)

For participants who attended slimming clubs (4, 5 and 6), the educational aspects of these experiences were transformative, and challenged participants’ understandings of their behaviours, promoting new, conscious, eating and lifestyle decisions:

“*It* (success) *was about education, because he and Anna and the others were educating us and making us think about what we were putting in our bodies, when we were putting it in our bodies, whether it was good, bad, ugly”* (6)

#### Exercise

Regular exercise was revealed to be important for 7 of the participants (1, 2, 3, 4, 6–8). Six of the participants (1, 2, 4, 6–8) exercised regularly. One participant (3) was recovering from knee surgery and was not exercising at the time of the interviews, but recognised that exercise was important to him previously. One participant explained that she was not physically active (5). The role of exercise in the participants’ weight-loss efforts was varied, but was not specific to weight loss or weight-loss maintenance, and many (*n* = 5) participants expressed that they felt that exercise was a fundamental component of their experience:

*“So it’s like I can’t, I find it really hard to diet without exercising*” (1)

For participants that exercised regularly, exercise reinforced dietary behaviours, and either prompted them to maintain dietary compliance, or was an underlying factor that made them eat for weight-loss purposes:

“*For me I think if I’m exercising three or four times a week, that punctuates a reminder to stay on track and eat clean or eat healthy and make appropriate choices”* (2)

“*But if I exercise I eat better. If I don’t exercise I eat worse*” (3)

While reflecting on how and why exercise was important, one participant revealed that if he drifted from his weight-loss diet he felt that time spent exercising was wasted, and so for this participant, exercise became a factor that prompted adherence:

*“My thinking is if I go out for a run, which I enjoy doing, and I eat badly, I’ve ruined that hour that I’ve spent going out for a run. You know, I’ve wasted that hour, whereas if I go out for a run and eat properly that hour of going out and running has been productive”* (3)

Other participants felt that undertaking exercise would allow them to be flexible with their eating behaviours, and would use exercise as a compensatory mechanism for overeating/drinking:

“*Friday morning I made myself get up and go and have a run first before I went, and that was kind of like cancelled it out, like for me in my head I could drink*” (7)

While motives for exercising was different for each of participant, for all that undertook exercise as part of their journeys, exercise appeared to add structure and re-affirmed behaviours. One participant also described how exercise prompted feelings of positivity, despite experiencing negative emotions as part of her weight-management journey:

*“Even times where you’re not feeling great because you’ve eaten too much or you’re not too happy with the way your weight is… if the gym’s going well at least that’s something that you’re able to do”.*(8)

For another participant (6), exercise, while perceived to be beneficial and important to his weight-loss endeavours, appeared to increase his appetite, highlighting that exercise was not universally beneficial:

*“You know, the more exercise I do the hungrier I become.”* (6)

#### Structure

Six of the participants stated that structure was a key component of their weight loss efforts (2, 3, 4, 6, 7 and 8), linking it to their perception of control:

*“Being in control is something I’m enjoying, and having structure is important.”* (2)

Creating structures by being organised and developing routines allowed the participants to manage their behaviours in the context of their environments, so that appropriate food was available when needed, that meal plans were pre-determined and adherent to their objectives. For some participants this was clearly defined and tangible (2, 3, 5, 6, 7 and 8):

“*I find that I work better with things written down and things to follow. So that’s probably why I find it easier to work within certain calories, because I know that’s how many calories I’m supposed to be having*” (8)

Other participants (1, 2 and 4) suggested that they had a less tangible structure, but that they recognised that routine was important to their successes:

*“I have a loose plan in place definitely. It’s a plan that I’ve fallen into I suppose through routine. I definitely know when I’m out of that routine”* (3)

#### Readiness to change

Six (3, 4, 5, 6, 7 and 8) of the participants explained that in order to succeed with their weight loss efforts that they needed to feel ready to change, and appeared to be an important factor underpinning weight loss specifically:

*“Well I think that there came a point at which I decided I needed to do something about it”* (6)

When asked to provide recommendations to others, some participants (2, 4 and 5) explained that readiness to change was paramount, recognising their readiness as a cognitive transformation:

“*You know, I think that my view about being ready to lose weight is the same about giving up smoking, and I gave up for two years. Before I gave up for two years I was absolutely ready psychologically to give up.”*(4)

#### Social support

An important sub-theme articulated by each participant was the role of social support, which appeared to be important for both weight-loss and weight-loss maintenance goals, and could come from sources such as spouses and work colleagues, to friends and slimming clubs:

*“And you can’t do a diet I don’t believe of any type unless you’ve got the support of those who live around you, the very close ones”* (6)

When asked about how they might advise others to lose weight, several participants (3, 4 and 6) suggested that dieters needed to make their weight-loss intentions public, and not try to lose weight on their own:

“*So you have to be you have to tell people that you’re doing it.”* (3)

“*I’d tell them not to do it on their own if they can avoid it”* (6)

Having supportive others provided the participants with stability and reassurance, and one participant (7) explained that she needed her support structures to help her when she was craving foods:

*“And like you say, her texting me the other day going I need chocolate, I need a pep talk, so it’s sort of like give each other a bit more encouragement”* (7)

While articulating the benefit of attending a slimming club, one participant (5) explained that eating to lose weight isolated her from family and friends, and that being part of a peer-group leant emotional support, providing her with a sense of belonging and solidarity:

“*Which is nice to feel that you are supported…You’re not the outcast. You’re not the odd one. You know, you’re part of a sort of team of people who are facing the same challenges together.*” (5)

#### Self-monitoring

All of the participants undertook self-monitoring activities to track their dietary intakes, body-weight, or body-size changes, particularly for weight-loss specific goals. The use of mobile technology and gadgets was common:

“*I use an app on my phone…I might track every meal that I have for four or five days. You know, I might just do a short food diary in effect just to see where I’m at and what I’m doing.*” (3)

While the use of such tools was important for some (1, 2, 3, 5, 7 and 8), subjective indicators such as the fit of clothing, feelings of energy and wellness, and the comments and affirmation of others were articulated to also be important indicators, regardless of whether weight loss or weight-loss maintenance was the primary goal at the time of interview. Interestingly, four of the participants (1–4) specifically cautioned against excessive monitoring however, suggesting that this could lead to obsessive behaviour, reflecting the weight-centeredness sub-theme (discussed earlier as a barrier):

*“I’d weigh myself before I went to the toilet, after I went to the toilet. You know, I was weighing myself several times a day, it became a little bit of an obsession.”* (4).

## Discussion

Participants in this research described weight loss and weight-loss maintenance as an omnipresent and on-going challenge. Weight loss appeared to be punctuated with successes and failures, and problems and difficulties were balanced and combated with behaviours and strategies that fostered adherence. The thematic framework (Fig. 1) demonstrates that the issues identified: dichotomous thinking, environments, social pressures and weight centeredness, were experienced as barriers to the participants’ weight-loss efforts, which at times stunted progress. By contrast, the facilitators: mindfulness, knowledge, exercise, structure, readiness to change, social support and self-monitoring, all assisted the participants’ weight-loss efforts, antagonistically. Some of these facilitators were meta-cognitive strategies (mindfulness), cognitive behavioural techniques (self-monitoring), motivational states (readiness to change), and environmental (social support) and educational (knowledge) strategies that participants experienced, developed or adapted to achieve their goals.

According to the theory of planned behaviour [19], if the balance between perceived barriers and facilitators of a behaviour change is biased towards facilitators, then the likelihood of lasting behaviour change is greater than if more barriers are perceived than facilitators. The participants in this study identified more facilitators than barriers, possibly because four of the participants were in a weight-maintenance phase having already achieved and experienced weight loss, and that the rest of the participants were experiencing weight losses at the time of the interviews. Participants in this research, therefore, had or were achieving weight-loss success at the time of the interviews. According to social cognitive theory (SCT) [20], the participant’s perceived/actual successes are likely to have increased their weight-loss self-efficacy, influencing the findings of this study [20, 21]; within SCT a person with high self-efficacy for lifestyle change is more likely to identify facilitators of change than barriers to change [20, 21]. Interestingly, Burke et al. [22] and Hammarström et al. [23] found strong emphases on barriers in their research (these studies explored the experiences of a self-monitoring intervention and the experiences of obese females aligned to an intervention study), perhaps because participants had failed in previous weight-loss attempts and possessed a lack of self-efficacy to achieve their weight-loss goals [21]. Indeed, high self-efficacy appears to be associated with long-term, successful weight management [1, 6], and has been linked to successful weight-loss and weight-loss maintenance in empirical research elsewhere [24].

It is interesting to note that participants here articulated a mostly positive weight-loss experience, especially considering that large proportions of dieters are unsuccessful in achieving their weight-management goals [5], and that the participants were not obtained from intervention research. Indeed, participants in this study self-initiated their weight loss endeavours, and so the experiences discussed in this research represent those obtained outside of an artificial research framework. It is possible, therefore, because participants self-initiated their weight management, that these participants possessed sufficient self-efficacy to develop the behaviours necessary to experience positive weight changes [21]. Within SCT behaviours are learnt through observational learning and modelling [20, 21]. Self-efficacy therefore can be enhanced by helping individuals learn and model new behaviours, or, by modifying unwanted behaviours by changing the reinforcements of that behaviour [21]. Strategies that enhance an individual’s weight-loss self-efficacy, such as those highlighted above, might be an effective weight-loss treatment [24].

Dichotomous/polarised thinking and behavioural patterns were an important experience encountered by each participant in this research. The literature recognises dichotomous/polarised thinking as a cognitive distortion where individuals view stark contrasts with no middle-ground, and is a thinking style linked to perfectionism and anxiety [25]. Individuals with dichotomous thinking might interpret not achieving a weight-loss goal as evidence of absolute failure, and are less likely to maintain weight-loss-related behaviours due to a perceived lack of success [25]. Rigid restraint is the concept that individuals possess overly restrictive eating habits and is an example of dichotomous/polarised thinking common to dieters [1, 6, 25–27]. When self-imposed eating restrictions become compromised, rigid restraint might then promote disinhibited eating, negative emotions, feelings of failure and the desertion of weight-loss dieting [27]. Participants in this research reported incidences of dichotomous thinking and rigid restraint, reflecting findings of similar research aligned to intervention studies [8, 22, 23]. Participants in this research, however, suggested that mitigating rigid restraint (achieved via metacognitive strategies such as mindfulness) reduced dichotomous thinking and disinhibited eating episodes, and improved emotional wellbeing and adherence. Indeed, participants in this research articulated that successful weight loss could be a transformative experience, where newfound behaviours and lifestyles are developed and reinforced by changes to cognitions (mindfulness), behaviour (self-monitoring), and environments (social support), synergistically. Treatment strategies such as cognitive-behavioural therapy [28] and mindfulness-based interventions [29] might be useful tools to assist with the development of similar cognitive-behavioural changes, and foster improved weight loss for some individuals.

Environmental issues created problems for the group, and these ranged from work-related and lifestyle constraints, to the exposure to appetite-promoting stimuli in the home. Environmental stability appears to be important for long-term weight management [6, 30], and participants suggested that when stability became compromised, through issues such as erratic working hours, travel, poor food availability and scheduling problems, that consistency to weight-loss behaviours became challenging. This reflects the theory of planned behaviour (TPB) [19], where life circumstances outside of an individual’s perceived behavioural control might create difficulty achieving or maintaining a behaviour (such as weight-loss dieting), despite the presence of the intention to engage with the behaviour [19]. Stressful life events were revealed to be particularly problematic by participants in this research, and stress-related and emotional eating episodes manifested from difficult life circumstances. Research elsewhere highlights similar findings [10], and multiple sources of evidence indicate that successful dieters develop coping strategies that accommodate for difficult life circumstances [1, 6–8, 10, 22, 23]. The ability to cope and successfully navigate difficult life events might therefore be an important factor in successful weight loss, regardless of the research context underpinning its observation [6].

Social difficulties were encountered by all participants in this research. Losing weight fostered alienation for some, where newfound weight-loss behaviours alienated individuals from valued peer and friendship groups, particularly during social activities, reflecting research elsewhere [7, 31]. Eating out led to the perception that participants needed to make eating decisions that lead to the consumption of non-diet foods, or risk alienation from their social groups. This led to some participants’ self-imposed social exclusion, which might be a common occurrence for some dieting individuals [7, 31, 32]. Social eating and drinking also exposed participants to stigma, where participants felt judged while eating out, which then led to feelings of self-consciousness, exacerbating the (perceived) need for isolation further. Participants were vocal of the need to obtain social support to accommodate such issues and eliminate feelings of alienation, provide stability, and engender the perception of moral support. Social support was therefore sought from friends, family and spouses, and from work colleagues and slimming clubs, which reflects evidence elsewhere [1, 6, 7, 26]. The perception of being supported appeared to be more important than the mode of support experienced however, which is complicit with evidence elsewhere [7, 33]. However, spouses and family could also act as saboteurs to participants’ efforts, tempting them with forbidden foods, or eating forbidden foods in their presence, with little consideration to the participants’ challenges, emotions and motivations. Interestingly, these findings have also been reported elsewhere [7, 34], and highlight that while significant others appear to play an important, facilitative role in dieting [1, 6, 7], they can be destructive also. Further research might be needed to corroborate some of the findings of this study and further investigate social difficulties experienced while dieting, in particular the motives and mechanisms of conscious/unconscious spousal sabotage, which appeared to be particularly challenging for participants in this study, but might also be understated in the literature at this time [7, 34].

Mindfulness was revealed to be a key facilitator of the participant’s efforts, which was a meta-cognitive transformation that engendered participants’ self-awareness, flexible restraint and self-regulation. Mindfulness, in this research, reflects Newman’s health as expanding consciousness theory, which suggests that lifestyle transformations occur through critical self-reflection and self-discovery, leading to an expanded consciousness, improved self-awareness, and greater self-control [35]. Mindfulness might also reflect the heightened vigilance articulated by participants in similar research aligned to intervention studies [7, 10, 36], where individuals experienced an intensified awareness of internal and external influences which challenge weight-loss consistency. While mindfulness was beneficial to participants’ efforts in this research, attentiveness to weight-loss behaviours could also be perceived negatively: careful decisions about eating and lifestyle had to be made and evaluated constantly, creating the perception of a weight-centred existence. The need to be constantly mindful and recommit to weight loss was therefore revealed to be emotionally challenging by some participants, especially in the presence of negative life events and difficult life circumstances. This is perhaps a novel and important finding of this research, and highlights that while attentiveness to weight loss was an important facilitator of success, this metacognitive strategy might be emotionally difficult to maintain, divorced from participants’ habituated cognitions and behaviours, and in some instances, promotes weight centeredness and obsessive behaviour towards achieving weight-loss goals. Indeed, weight centeredness reflected, and was the consequence of, an all-encompassing and difficult weight-loss journey.

Participants suggested that improving knowledge enhanced autonomy and led to informed decision making, assisting weight-loss efforts. Research elsewhere has found similar findings [37], and increasing practical knowledge of food and recipes, and theoretical knowledge such as energy balance and nutrition, might assist weight-loss efforts, reflecting SCT and TPB [19–21]. Within TPB, perceived behavioural control (which along with intention to engage with behaviour leads to the development of that behaviour) is analogous to self-efficacy [19–21], is an individual’s confidence in their ability to perform/achieve a desired behaviour, and is influenced by resources and opportunities [19]. Improving knowledge might enhance an individual’s perceived behavioural control by engendering the perception that they have greater knowledge to successfully undertake/complete the behaviour [19]. Interestingly, one participant in this research articulated reservations about furthering their understanding of nutrition science as an aide to weight loss however. For this participant, understanding the scientific underpinning of nutrition depersonalised eating and led to confusion and exposure to conflicting information about appropriate dietary choices, conflicting with the hypothesis above.

Exercise was also found to play an important, beneficial and multi-faceted role in this research. Regular exercise reinforced dietary behaviours, was used as a tool to promote and enhance flexible restraint, and was a potent modifier of mood and self-esteem. Importantly, exercise appeared to enhance self-regulatory behaviours, which appears to be consistent with empirical data [10]. Exercise also provided structure and routine, and becoming organised and developing structure was a formative experience that led to the perception of a greater internal locus of control, which was clearly articulated by participants here, and has been discussed in literature elsewhere [1, 6, 26, 38, 39]. While exercise was discussed as being unequivocally beneficial by participants, exercise was also revealed to have appetite-promoting effects by some, making dietary compliance challenging at times. Indeed, empirical data indicates that exercise might have appetite-promoting or appetite-reducing effects depending on its mode and intensity [40], supporting this observation. This is perhaps the first qualitative study that highlights exercise’s appetite-modifying effects within a naturalistic sample of participants engaged with weight-loss dieting, and might indicate that the prescription of exercise interventions within weight-loss contexts might need to be tailored towards its appetite-reducing effects for some individuals.

Self-monitoring appears to be widely associated with successful weight management [1, 6, 22], was reported to be a facilitator of weight loss by participants in this research, and those in intervention-based experiential research elsewhere [8, 22, 23]. Participants tracked dietary intakes using electronic tools and devices, but also monitored exercise data and valued qualitative assessments of wellbeing. Participants explained that they used smart phone apps to complete food and exercise diaries to collect and tabulate data that they could use to monitor and assess their progress. Monitoring and feedback appear to be important behaviour-change techniques [41], and participants in this research made use of mobile technology and online tools to assist their utilisation for weight-loss purposes. For participants in this research, and those in similar studies elsewhere [8, 22, 23], consistent self-monitoring appeared to be important aspect of a successful weight-loss experience. However, despite the broadly positive role articulated, some participants also explained that self-monitoring might also promote obsessiveness (about maintaining the behaviour), exacerbate dichotomous thinking (if results are not achieved in-line with expectations), and lead to weight-centeredness (if regular weighing and body measurements are the self-monitoring activities of choice), which were all unanimously described to be damaging by participants. Indeed, an important finding of this research is the need to mitigate the detrimental effects of the preceding factors, and that some of the techniques and strategies employed/developed by participants, such as self-monitoring and mindfulness, might exacerbate their effects in some contexts. This offers an important counterargument to the utilisation of such techniques as weight-loss interventions in some dieting individuals, and partially reflects Burke and colleagues findings that self-monitoring interventions might not be universally agreeable [22], despite its widespread acclaim within literature [1, 6–8, 36].

### Study limitations

While weight loss was revealed to be a complex problem, where physical, environmental, social and behavioural factors disrupt and assist weight loss, the homogeneity of the sample necessitates that further research might be needed to gain a broader insight into the weight-loss experience. Indeed, participants were recruited to this study from local slimming clubs, gyms and health clubs, and through colleagues’ networks, using iterative sampling techniques. This mode of recruitment might have been insufficient to recruit participants from disadvantaged and black and minority ethnic backgrounds, who might not have access to these establishments. A lack of diversity within the sample might mean that information relevant to the experiences of all social and ethnic groups is not fully-represented in this study. The sample of this research was, however, sufficient to achieve data saturation, and this study therefore provides useful, in-depth information about living weight-loss experiences. Further research is required to explore the issues identified within this study in depth, and within wider social contexts.

### Study strengths

This study explored real-life weight-loss experiences, as opposed to experiences captured within research environments. This study, therefore, offers information about the experiences of individuals who initiated and undertook weight loss in real-life contexts outside of a research framework, highlighting the barriers and facilitators that they experienced. The findings of this study offer new information about the weight-loss experience, and should serve as a catalyst for further investigation.

## Conclusion

The results of this study highlighted that for participants in this research, weight loss was experienced as a difficult and enduring journey, with physical, cognitive, behavioural, social and environmental dimensions. Weight loss was, therefore, a challenging journey that was punctuated with factors and experiences that either assist or disrupt the endeavour. Weight loss was assisted by mindfulness, knowledge, exercise, structure, readiness to change, social support and self-monitoring. Weight loss was challenged by dichotomous thinking, environments, social pressures and weight centeredness. A deeper understanding of the breadth, complexity and interaction of factors implicit to weight management is essential for public health promotion, for educators, and for policy makers similarly. Therefore, on the basis of this study, future research should look to investigate the effects of social alienation and spousal sabotage experienced as a consequence of weight-loss dieting, in a bid to further the development of interventions that ameliorate their effects. Similarly, the incorporation of flexible weight-management behaviours, based on cognitive-behavioural strategies which mitigate the perceptions of weight centeredness, dichotomous thinking and obsessive behaviour, which were identified as major challenges in this research, merit further investigation as potential weight-loss treatments.

## Ethics approval and consent to participate

Institutional ethical approval was granted for this study by Sheffield Hallam University’s research degree’s ethics committee. All participants provided informed consent to undertake this research, which was performed in accordance with the declaration of Helsinki.

## Consent for publication

Consent for the publication of data was provided by participants.

## Availability of data and materials

The dataset supporting the conclusions of this article will not be shared publicly, due to information contained within the interviews that could be linked to participants. Individual requests for data can be made to the corresponding author however.

## Abbreviations

SCT: social cognitive theory
TPB: theory of planned behaviour
